# Physicochemical Characterization and Simulation of the Solid–Liquid Equilibrium Phase Diagram of Terpene-Based Eutectic Solvent Systems

**DOI:** 10.3390/molecules26061801

**Published:** 2021-03-23

**Authors:** Maha M. Abdallah, Simon Müller, Andrés González de Castilla, Pavel Gurikov, Ana A. Matias, Maria do Rosário Bronze, Naiara Fernández

**Affiliations:** 1 iBET, Instituto de Biologia Experimental e Tecnológica, Apartado 12, 2781-901 Oeiras, Portugal; maha.abdallah@ibet.pt (M.M.A.); amatias@ibet.pt (A.A.M.); mbronze@ibet.pt (M.d.R.B.); 2 TQB-NOVA, Instituto de Tecnologia Química e Biológica António Xavier, Universidade Nova de Lisboa, Avenida da República, 2780-157 Oeiras, Portugal; 3 Institute for Thermal Separation Processes, Hamburg University of Technology, Eißendorfer Straße 38, 21073 Hamburg, Germany; simon.mueller@tuhh.de (S.M.); andres.gonzalez.de.castilla@tuhh.de (A.G.d.C.); 4 Laboratory for Development and Modelling of Novel Nanoporous Materials, Hamburg University of Technology, Eißendorfer Str. 38, 21073 Hamburg, Germany; pavel.gurikov@tuhh.de; 5 FFULisboa, Faculty of Pharmacy, University of Lisbon, Avenida Professor Gama Pinto, 1649-003 Lisbon, Portugal

**Keywords:** deep eutectic solvents, terpenes, physical characterization, simulation, equilibrium phase diagram, chemical interactions

## Abstract

The characterization of terpene-based eutectic solvent systems is performed to describe their solid–liquid phase transitions. Physical properties are measured experimentally and compared to computed correlations for deep eutectic solvents (DES) and the percentage relative error *e*_r_ for the density, surface tension, and refractive index is obtained. The thermodynamic parameters, including the degradation, glass transition and crystallization temperatures, are measured using DSC and TGA. Based on these data, the solid–liquid equilibrium phase diagrams are calculated for the ideal case and predictions are made using the semi-predictive UNIFAC and the predictive COSMO RS models, the latter with two different parametrization levels. For each system, the ideal, experimental, and predicted eutectic points are obtained. The deviation from ideality is observed experimentally and using the thermodynamic models for Thymol:Borneol and Thymol:Camphor. In contrast, a negative deviation is observed only experimentally for Menthol:Borneol and Menthol:Camphor. Moreover, the chemical interactions are analyzed using FTIR and ^1^H-NMR to study the intermolecular hydrogen bonding in the systems.

## 1. Introduction

Deep eutectic solvents (DES) have been widely investigated as new alternatives and analogues to ionic liquids. In many cases, they are more eco-friendly and cheaper alternatives to ionic liquids and conventional solvents. They are prepared by combining a hydrogen bond donor and hydrogen bond acceptor near the eutectic point (eutectic temperature and molar ratio) at a temperature above the melting temperature of the homogenous mixture formed. The resulting system should have a considerably lower melting point compared to its ideal eutectic and the melting point of its individual constituents [1,2,3] as they are characterized by the presence of hydrogen bonding and strong non-ideal attractive interactions [4,5].

The use of non-toxic compounds has been highly investigated for the preparation of DES. Terpenes are produced by living organisms, including bacteria, fungi, algae, and plants. They comprehend a chemical space of approximately 30,000 secondary metabolites that are divided into isoprene units and differ in the role and structure. The monoterpenes (C_10_, two isoprene units) and sesquiterpenes (C_15_, three isoprene units) are the more volatile groups and are formed in vegetation [6,7]. Monoterpenes include menthol, thymol, linalool, borneol, eucalyptol, camphor, 1,8-cineole, α-pinene, limonene, and citral. They are found in essential oils of different plants, such as mentha, thyme, lemon, juniper, lavender, eucalyptus, marjoram, rosemary, pine, salvi, and geranium among others [8]. This class of compounds have been widely used in pharmaceutical applications for the development of drugs due to their bioactive properties. For instance, menthol and borneol have been employed in ocular applications, including the dry eye disease [9,10,11,12]. Thymol has been employed as skin permeation enhancer of the drug meloxicam [13]. Furthermore, ibuprofen has been combined with terpene eutectic systems to increase its transdermal permeation [14].

In order to check whether the mixture in question is a DES, the thermodynamic behavior should be strongly non-ideal. For this test, the simulation of the solid–liquid equilibrium (SLE) phase diagram of the two solids is done. An eutectic mixture can be considered ‘deep’ when the real eutectic temperature is significantly lower than the ideal one (*T*_E, real_ < *T*_E, ideal_) [15,16,17].

In this work, eutectic mixtures are prepared by combining four terpenes shown in Table 1, to prepare the eutectic systems menthol:borneol (Men:Bor), thymol:borneol (Thy:Bor), menthol:camphor (Men:Cam), and thymol:camphor (Thy:Cam). The physical properties such as melting temperature, viscosity, and density are obtained experimentally. The refractive index and surface tension are computed using empirical correlations developed to estimate the properties of the eutectic mixtures [18,19]. Furthermore, the thermodynamic properties are obtained to calculate the SLE phase diagram, from which the eutectic molar composition and temperature can be determined. In addition, the chemical properties of the prepared DES are studied to assess the structure and the presence of hydrogen bonding. The SLE phase diagrams are calculated using the UNIversal Functional Activity Coefficient (UNIFAC) [20] and the Conductor-like Screening Model for Real Solvents (COSMO-RS) [21,22].

## 2. Results and Discussion

### 2.1. Physical Properties of the Eutectic Systems

The physical parameters obtained theoretically and experimentally for a temperature of 298 K are shown in Table 2. The eutectic molar ratio at which the systems are synthesized and analyzed is obtained according to the ideal solid–liquid equilibrium phase diagram, which is computed using the thermodynamic properties of the compounds involved in the system (shown in Section 2.4.). The computed critical properties of the eutectic systems are shown in Tables S2 and S3 of the Supplementary Material. The viscosity and density are important physical properties required for the design and optimization of various chemical processes employing the solvents, such as heat exchangers, separation units and agitation equipment, among others [23,24]. The binary system Men:Bor (128.07 mPa/s) is shown to be more viscous than the other systems, followed by Thy:Bor (45.47 mPa·s). Thus, the eutectic solvents with borneol in the system have a higher viscosity than those with camphor. The density obtained experimentally is shown to be between 0.9152 to 0.9717 g/mL for the different systems. These values are lower than the values predicted using the models proposed in the literature. The percentage relative error *e*_r_ is computed in order to assess the accuracy of the computations using the models in comparison to the experimental results. The value is shown to lie between 17.3% to 21.5%. Based on the previous works, the values for the density are shown to be similar as the values range from −13% to 22% [25].

Moreover, the surface tension is a physical property that describes the inclination of a fluid, which defines its minimal surface area. It plays a vital role in the permeability of the solvent and the design of processes. Its value increases with the decrease of the temperature and the kinetic molecular energy of the liquid [19,26]. It is shown to be in the range of 29.04 to 31.75 mN/m for the solvent systems. The computed values are shown to be similar to the experimental values, as the *e*_r_ values ranging from −20.96% to 6.25%, as shown in Table 3. These values are in correlation with previous works that have shown that most of the investigated systems have a surface tension value lower than 50 mN/m [19].

The refractive index of the eutectic solvent systems has been investigated as a useful tool to identify compounds, assess the purities of substances and verify the concentrations of mixtures [27,28]. It is a fundamental parameter to understand the intermolecular interactions and the behavior in solutions [29]. The refractive index values of the eutectic mixtures are shown to be in the range of 1.4635 to 1.5105, which are in accordance with previous studies [18]. The experimental refractive index results are similar to the estimated values using the models as the *e*_r_ for this parameter is the lowest, ranging from −2.05% to 0.25%, as shown in Table 3. Thus, based on the results obtained for the study of the four terpene eutectic mixtures, the proposed models are mostly fit for the computation of the refractive index, as a lower error is obtained in comparison to the other properties.

### 2.2. FTIR and ^1^H NMR Analysis

**Figure 1:**
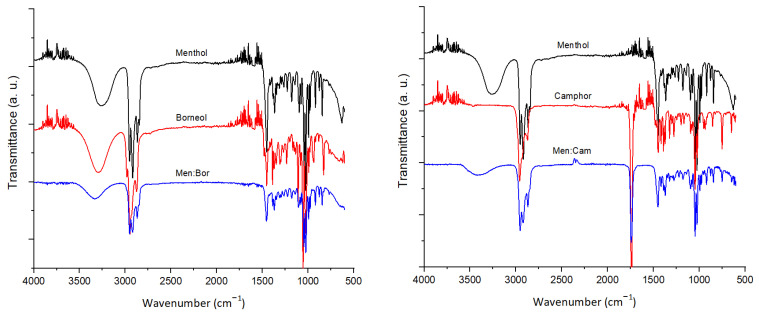

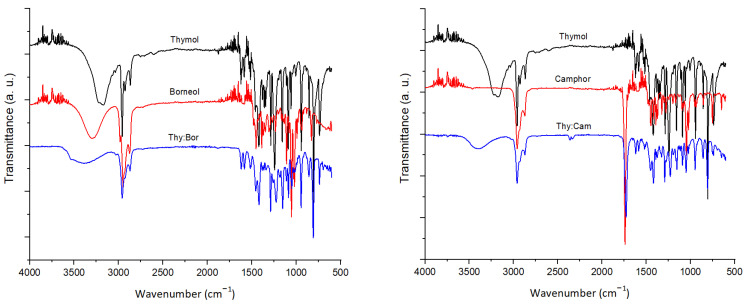
FTIR spectra of the pure terpenes and eutectic systems.

The eutectic mixtures and the pure components used to prepare them are analyzed using FTIR to assess their chemical structure, as shown in Figure 1. Menthol, thymol, and borneol are terpene alcohols and exhibited an O−H stretching vibration at 3270, 3170, and 3300 cm^−1^, respectively. In contrast, camphor displayed a strong absorption band at 1739 cm^−1^, which is a characteristic of ketones having a C=O stretching vibration. Furthermore, symmetrical and asymmetrical −CH_2_ stretching vibrations correspond to the bands at 2958 and 2867 cm^−1^, respectively. These bands are superimposed upon the O−H stretching. These terpene molecules present a hydrogen bond between the oxygen atoms and the hydroxyl groups. The O−H stretching vibration peaks shifted to higher wavenumbers in comparison to the pure compounds due to the hydrogen bonding that took place through the O−H groups [30].

**Figure 2:**
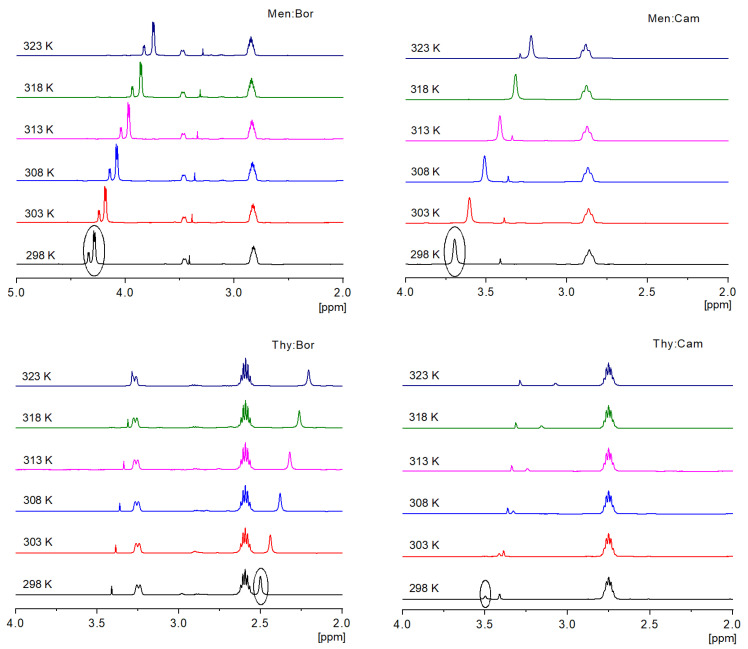
Magnified ^1^H-NMR spectra of the eutectic systems as a function of temperature.

**Figure 3:**
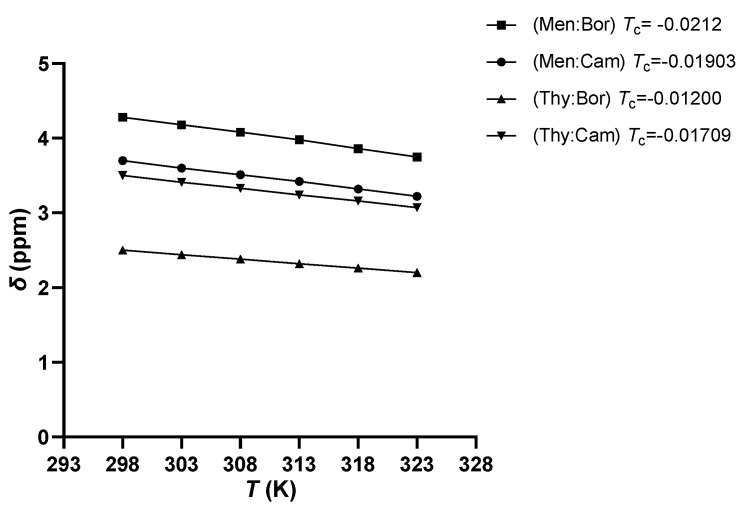
Plot of the chemical shifts (*δ*) of the OH proton as a function of temperature for each eutectic system.

Furthermore, the eutectic mixtures and the pure components are analyzed using ^1^H-NMR. The temperatures measured lie between 298 to 323 K at an increment of 5 K to study the chemical shifts that take place due to the H-bonded protons for the inter- and intramolecular bonds [31,32]. It has been shown that as the temperature of the sample is increased, a decrease in the intermolecular bonding takes place, which leads to an upfield shift of the H-bonded proton [33], as shown in Figure 2. A temperature coefficient (*T*_coeff_) is obtained to study the magnitude of the shift and the type of H-bonding associated. This coefficient is calculated as the slope of the linear correlation of the chemical shifts (δ) as a function of the temperature, as shown in Figure 3. In nonpolar solvents, a *T*_coeff_ value more negative than −0.005 ppm/K indicate intermolecular H-bonding takes place, while a *T*_coeff_ value less negative than −0.003 ppm/K indicate intramolecular H-bonding [34]. In Figure 2, the upfield shift of each –OH functional group is present and labeled in each system as the temperature is increased. Figure 3 shows that the Tc values obtained for each system range between −0.0212 to −0.012 ppm/K, suggesting an intermolecular H-bond is happening. As for the pure terpenes, an upfield shift of the –OH functional group is also observed when the temperature is increased. However, the extent of the chemical shift is lower than that observed in the eutectic systems, as *T_c_*_oeff_ ranges between −0.004 to −0.006 ppm/K, as shown in Table S5. Thus, the molecular interaction taking place in the pure terpenes differs from that in the eutectic systems. This suggests that significant hydrogen bonding occurs between two different molecules, hence the eutectic systems do not behave as an ideal mixture.

### 2.3. TGA and DSC Analysis

The thermodynamic properties of the pure compounds studied are measured in order to calculate the SLE phase diagram. Table 4 displays the values obtained using DSC that are used for the SLE phase diagram calculations. The degradation temperature *T*_d_ of the DES is obtained using TGA to assess the limit of the heating temperature of the eutectic systems using DSC. The glass transition (*T*_g_) and crystallization temperatures (*T*_cr_), and the enthalpy of crystallization (Δ*H*_cr_) are obtained for each eutectic solvent system at a molar ratio closest to the eutectic point, as shown in Table 5. A remarkable difference in the enthalpy of crystallization is observed between the menthol- and thymol-based systems, as the latter have a much lower observed value. Crystallization is affected by the molecular nature of the substances and the kinetics of the cooling process and the rate of the temperature decrease in DSC affects the crystallization of the molecules [35,36]. For instance, during crystallization at low temperatures, inefficient crystal packing could form and in order to build a crystal lattice, molecules must adapt a proper conformation which could be hindered at low temperatures [35,37,38]. Hence, an attraction between unlike molecules as well as the inefficient packing in the crystal structure lead to the formation of the glassy states. Consequently, in the studied eutectic systems, the formation of the glassy state at a low temperature occurs due to a strong negative deviation from ideality as a result of an interaction between unlike molecules [35].

Based on the results obtained using DSC, the thermal transition behavior of the eutectic systems can be described by cooling to a glass state at low temperature. The *T*_g_ is obtained for each system and upon heating, crystallization takes place and a *T*_cr_ is recorded above the *T*_g_, as shown in Table 5. Similar behavior was observed in another study when assessing the thermodynamic behavior of eutectic solvent systems [35,39].

### 2.4. SLE Phase Diagram Simulation

**Figure 4:**
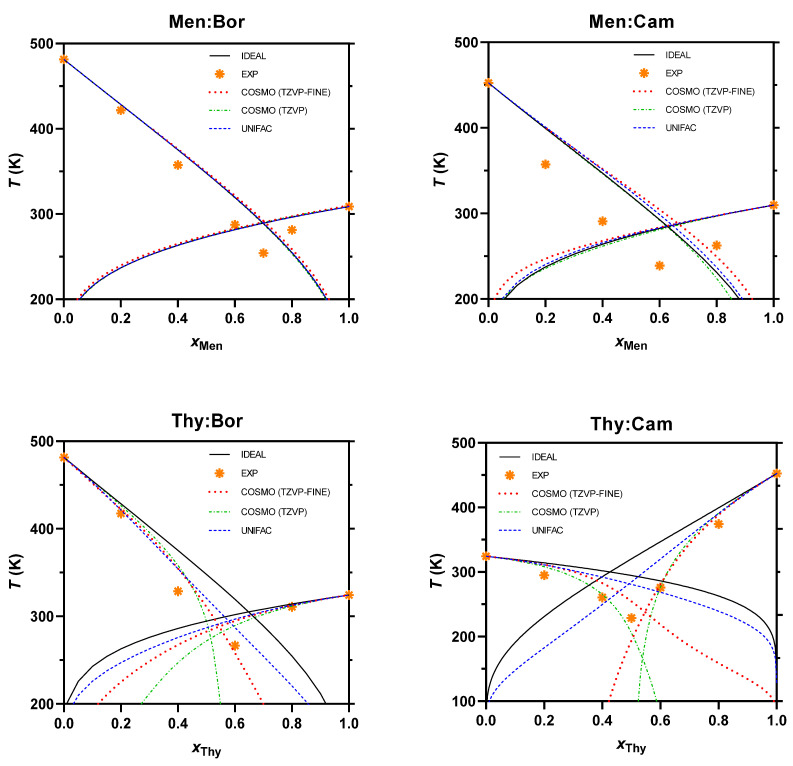
Measured SLE phase diagrams of the terpene-based solvent systems.

The SLE phase diagrams are obtained based on Equation (6). The values of *T*_fus_ and Δ*H*_fus_ for the pure components that are used to simulate the systems are shown in Table 4. For the real systems, the diagrams are computed using the COSMO-RS and UNIFAC models, as shown in Figure 4 along with results of DSC experiments. Assuming that the calculations are acceptably accurate [5,40], from these plots, the eutectic temperatures and molar ratios at the eutectic point can be estimated (Table 6). 

For the menthol-based solvent systems, a low extent in the negative deviation from ideality is observed in the SLE diagrams. The UNIFAC and COSMO-RS models estimate the behavior to be comparable to the ideal eutectic. For instance, the activity coefficient ranges from 1 to 1.01 and from 1.02 to 1.16 for Men:Bor based on the UNIFAC and COSMO-RS (TZVPD-FINE) models, respectively. As menthol is a terpene alcohol with the –OH group attached to a cyclic ring and the components are a mixture of terpene alcohols having similar functional groups, the predicted activity coefficient is close to ideality. Thus, the ideal eutectic temperatures of Men:Bor and Men:Cam (289.8 and 284.7 K respectively), are very close to the predicted real eutectic temperatures (289.8 to 290.7 K and 283.4 to 288.1 K for Men:Bor and Men:Cam, respectively). The models do not predict a negative deviation from ideality similar to the one observed in the experimental results. For Men:Bor, a lower negative deviation is observed in comparison to Men:Cam and the thymol-based systems. Nevertheless, it cannot be confirmed that the behavior has such clear negative deviation from ideality for both systems. 

For the thymol-based solvent systems, the eutectic temperatures obtained experimentally and using the models are lower than the ideal ones. For Thy:Bor, the estimated real eutectic temperatures are 274.4, 293.2, and 300.8 K obtained using COSMO-RS (TZVP and TZVPD-FINE levels) and UNIFAC, respectively, which are lower than the ideal one (309.6 K). A similar behavior is observed for Thy:Cam as a negative deviation is shown from ideality (300.1 K) to the estimated real behavior (from 166.2 to 281.7 K). The experimental SLE measurements display a significant negative deviation from ideality, which is also correctly predicted by the models. Thus, these systems could be considered as DES. 

By comparing the estimated real SLE using the models, it can be shown that the COSMO RS model predicts eutectic points further away from ideality than the UNIFAC model, and a higher deviation is observed in the COSMO-RS parametrization level TZVP than that of the TZVPD-FINE level. Moreover, the plots of the thymol-based solvent systems show that the models estimate a deviation of the eutectic concentration. For example for Thy:Bor, the ideal eutectic concentration is 0.72 while the estimated value by the models is 0.65, 0.52, and 0.58 for UNIFAC, COSMO-RS (TZVP and TZVPD-FINE levels), respectively. This shows that the structural interaction is not fully described by the models and that additional effects may be taking place.

## 3. Materials and Methods

### 3.1. Materials

The chemicals used were the following: DL-menthol (CAS [89-78-1], ≥95%) and DL-camphor (CAS [76-22-2], ≥96%)) from Sigma-Aldrich (St. Louis, MO, USA), thymol (CAS [89-83-8], ≥98%) and L-borneol (CAS [464-45-9], ≥97%)) from Alfa Aesar (Haverhill, MA, USA). Deuterated dimethyl sulfoxide (DMSO-d6, CAS [67-68-5]) and chloroform (CDCl3, CAS [865-49-6]) and tetramethylsilane (TMS, CAS [75-76-3]) used in NMR experiments was purchased from Sigma-Aldrich (St. Louis, MO, USA).

### 3.2. Eutectic Mixtures Preparation

To prepare the eutectic mixtures, the pure terpene components are mixed at the chosen concentration, magnetically stirred and heated at 90 °C until a homogenous transparent liquid system is obtained. 

### 3.3. Analysis of the Physical Properties

The viscosity and density of the mixtures close to the eutectic point are obtained experimentally using an Anton Paar viscometer (SVM 3001, Graz Austria) in a range of temperatures between 293 and 323 K. The temperature reproducibility is 0.03 K. The measurements are performed in triplicates for each sample.

To measure the surface tension experimentally, a KSV Attension tensiometer (KSV Sigma 702) and platinum–iridium Du Noüy ring method are employed for surface and interfacial measurements. The ring height, diameter and wire thickness are 25, 18.7, and 0.37 mm, respectively. The measurements are done at a temperature of 298 K in a thermostat bath (Lab Companion RM0525G). Three replicates are done for each mixture close to the eutectic point. An Abbe refractometer is used to determine the refractive index of the eutectic mixtures using natural light.

A theoretical computation of the physical properties is done to assess the density (*ρ*_L_), surface tension (*σ*), and the refractive index (*n*) were estimated using empirical models.

The density *ρ*_L_ (g/mL) and is computed based on Equation (1) [25].

(1) $$\rho_{L}=-1.13\times 10^{-6}T_{c}^{2}+2.566\times 10^{-3}T_{c}+0.2376\omega^{0.2211}-4.67\times 10^{-4}V_{c}-\;4.64\times 10^{-4}T,$$

where *T* is the temperature (K) and *ω*, *V*_c_ and *T*_c_ are the acentric factor, critical molar volume (cm^3^/mol) and temperature (K), respectively. In order to assess these parameters, the critical properties of the compounds were obtained based on the Modified Lydersen and Joback–Reid model [41,42], as shown in Equations (S1) to (S11) of the Supplementary Material.

For the surface tension and the refractive index, empirical models that have been proposed as universal approximations to estimate these properties for the eutectic systems are applied [18,19], as shown in Equations (2) and (3).

(2) $$\sigma_{L}=\sigma_{1}\ln\left(\rho \right)+\sigma_{2}\omega^{\frac{P_{c}}{P_{\mathrm{ref}}}}+\sigma_{3}T_{c}\ln\left(A_{1}\rho^{2}\left[V_{c}+\frac{\sigma_{4}A_{2}}{\omega^{2}}\right]\right)+\frac{\sigma_{5}A_{3}M_{w}\sqrt{T}}{P_{c}\ln\left(\frac{V_{c}\rho A_{4}}{\sqrt{T}}\right)}+\sigma_{6},$$

(3) $$n=A_{5}\omega^{3}+A_{6}\frac{\omega^{2}}{M_{w}}+A_{7}P_{c}+A_{8}+\frac{A_{9}\omega }{T},$$

where *M*_w_ is the molecular weight obtained according to Equation (4) for compounds *i* and *j*, *P*_c_ is the critical pressure (bar), *P*_ref_ is a reference pressure (1 bar), *σ*_1_–*σ*_6_ (mN/m) and *A*_1_–*A*_9_ are adjusted and optimized parameters for the proposed model and are shown in Table 7 [18,19].

(4) $$M_{w}=\frac{\left(x_{i}M_{w,\;i}+x_{j}M_{w,j}\right)}{x_{i}+x_{j}},$$

The percentage relative error $e_{r}$ is computed for the density, surface tension and refractive index according to the Equation (5).

(5) $$e_{r}=100\left(\frac{theoretical-experiemtal}{experimental}\right)$$

### 3.4. Fourier-Transform Infrared Spectroscopy Analysis

Fourier-transform infrared spectroscopy (FTIR) (Class 1 Laser Product Nicolet 6100, San Jose, CA) was used to assess the structure of the pure compounds as well as the prepared eutectic mixtures. FTIR absorption spectra are recorded with 4 cm^−1^ resolution and with 40 scans of the sample in the range 4000 to 600 cm^−1^. 

### 3.5. ^1^H-NMR Analysis

NEO500 spectrometer, Bruker, Rheinstetten, Germany, coupled to a temperature probe (BTO2000) is used to analyze the pure terpenes and the prepared eutectic mixtures. For the pure compounds, 5 mg of the solids are solved in 650 μL of CDCl_3_ with TMS in the NMR tube. Before acquisition, the samples are equilibrated for 15 min using a Thermocouple-T to adjust and monitor the temperature. Proton chemical shifts are calibrated in reference to TMS (0 ppm). To analyze the eutectic solvent systems, DMSO-d6 is placed in a sealed capillary tube inside the NMR tube containing 300 µL of the solvent systems, to avoid the interference of DMSO-d6 in the hydrogen bonding in the systems. Equilibration for 15 min to ensure the required temperature, and the samples are locked using the frequency of the DMSO-d6 (2.8 ppm). The spectra are recorded at 298 to 323 K for the pure compounds and 298 to 313 K for the eutectic systems, with an increment of 5 K. For both pure compounds and eutectic systems, the spectra are obtained at 500 MHz with a 30° pulse angle, 4.5 s pulse delay, and 16 scans.

### 3.6. TGA and DSC Analysis

The thermal degradation temperatures are determined using thermogravimetric analyzer TGA (TA instrument model TGA Q50). Samples are placed in the crucible under nitrogen atmosphere (flow rate of 50 mL/min) and heated up to 773 K at a rate of 2 K/min. 

The thermodynamic properties are obtained using differential scanning calorimetry (DSC) (TA Instrument model DSC Q200) under anhydrous high-purity nitrogen at 50 mL/min. Samples were sealed in aluminum pans. For borneol and camphor, the temperature is cooled to 193 K, then heated from 193 to 503 K, cooled to 193 K and heated back to 503 K at a rate of 6 K/min. For thymol, menthol, the same cycles, cooling and heating rates are applied with the cooling and heating temperatures of 193 and 333 K, respectively. The computed parameters are determined in the second reheating cycle. 

To obtain the experimental SLE, the eutectic mixture at different molar ratios are studied on DSC by cooling with a rate of 6 K/min conducted until 193 K. A heating cycle is done until a temperature that is approximately 10 K higher than the liquidus temperature of the sample. The sample is then cooled back to 193 K and reheated to the same temperature as in the first cycle. The DSC measurements are obtained in the third cycle.

### 3.7. SLE Phase Diagram Calculation

The SLE phase diagram of the solvent systems is plotted using COSMO-RS and UNIFAC in order to obtain the suitable range of the components molar ratio and the operating temperature at which these solvents could act as DES. Based on these plots, the suitable concentration of the components is specified at which all further studies are conducted. 

The shown SLE phase diagrams are based on the following simplified thermodynamic relation Equation (6), in which the less relevant change in calorific capacity (Δ*C_p_*) is neglected [43,44]

(6) $$\ln\left(x_{i}^{L}\gamma_{i}^{L}\right)=\frac{∆H_{fus}}{RT}\cdot \left(\frac{T}{T_{fus}}-1\right),$$

where $x_{i}^{L}$ and $\gamma_{i}^{L}$ are the molar ratio and the activity coefficient of component i, respectively. Δ*H_fus_* is the enthalpy of fusion (J/mol), *R* is the gas constant (8.314 J/mol·K), *T* and *T_fus_* are the temperature of the system and the melting temperature (K) of a pure component, respectively. The SLE simulation was done using two software applications: MATLAB and COSMOthermX19. The use of the two models is compared to the ideal SLE in which the activity coefficient is equal to unity.

#### 3.7.1. UNIFAC Function Computations

UNIFAC is based on the fragmentation of the molecules into their functional groups to let these groups interact with each other to calculate the activity coefficient $\gamma_{i}^{L}$ [45,46,47]. It is computed as a function of the combinatorial activity coefficients ($\gamma_{i}^{C}$) and residual activity coefficients ($\gamma_{i}^{R}$), as shown in Equation (7).

(7) $$\ln\left(\gamma_{i}^{L}\right)=\ln\left(\gamma_{i}^{C}\right)+\ln\left(\gamma_{i}^{R}\right),$$

The combinatorial activity coefficient depends on the size and spatial conformation of the molecules present in the system. On the other hand, the residual activity coefficient accounts for the energetic interactions obtained using the group activity coefficients of both the mixture and the pure substances. The equations for the computations to obtain both $\gamma_{i}^{C}$ and $\gamma_{i}^{R}$ are included in the supporting information [45,48].

The coefficient $\gamma_{i}^{C}$ is computed based on the Flory-Huggins expression and the Staverman–Guggenheim correction term [43,49]. Its computation, as well as the computation of the coefficient $\gamma_{i}^{R}$ are based on the Equation (S12) to (S22) in the Supplementary Materials. 

An own implementation of UNIFAC (on MATLAB 2019a) was used in this work. The parameters of UNIFAC were published by Hansen et al. (1991), Gmehling et al. (1993), and Oracz et al. (1996) [50,51,52].

#### 3.7.2. COSMO-RS Simulation

COSMO-RS is also used to calculate the SLE phase diagrams. It relies on quantum chemistry and statistical thermodynamics in order to predict the properties of the target fluid mixtures from first principles [22,53]. The activity coefficient is calculated by taking into consideration the surface charge of the molecules present calculated from quantum chemistry instead of its chemical groups. This allows for a much more predictive application of the model straight from the structure of the molecules even to molecules for which no parameters are available with UNIFAC [54]. The conformers of the terpene used were generated using COSMOconf software (version 3.0). The COSMOthermX19 parameterization was applied at the BP-TZVP and BP-TZVPD-FINE levels.

## 4. Conclusions

The characterization of the four different terpene-based eutectic solvent systems is done to obtain their physicochemical properties. The density, surface tension, and refractive index are computed experimentally and compared to models proposed in the literature for each eutectic system. The accuracy of the models was studied using the percentage relative error $e_{r}$ for each solvent system, which ranged from 17.3% to 21.5% for the density, from −21.0% to 6.3% for the surface tension and from −2.05% to 0.26% for the refractive index. Furthermore, the chemical structure and interactions are analyzed on the basis of FTIR and ^1^H-NMR measurements to confirm the presence of the intermolecular hydrogen bonding in the eutectic systems. The thermodynamic properties, such as the enthalpy of crystallization, the degradation, glass transition, and crystallization temperatures are measured using TGA and DSC. From these obtained values, the SLE phase diagram of the eutectic mixtures is studied using UNIFAC and COSMO-RS with two different parametrization levels. From the curves, the eutectic temperature and molar concentration is estimated for each system and the deviation from ideality is studied to assess the behavior of the systems checking if they behave as DES. The UNIFAC and COSMO-RS models predict comparable but not completely accurate curves in comparison to the experimental data. They show comparable results without any of the models capturing the trends better than the other in all cases studied here. However, optimization of the models is needed to improve the prediction of the interaction in the systems. The difference in the chemical interactions between the pure compounds and the eutectic systems, observed experimentally and analyzed by simulation, prove that in fact the systems do not act as an ideal mixture due to the presence of significant hydrogen bonding, which confirms their behavior as possible DES candidates. 

## Figures and Tables

**Table 1:** Chemical structure of the terpenes.

Compound	Chemical Structure
Menthol	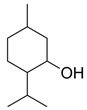
Thymol	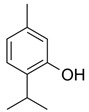
Borneol	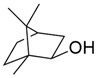
Camphor	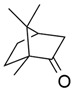

**Table 2:** Experimental and theoretical properties of the eutectic systems.

		Eutectic Systems			
		Men:Bor (7:3)	Men:Cam (3:2)	Thy:Bor (7:3)	Thy:Cam (1:1)
*M*_W_ (g/mol)		155.66	154.65	151.43	151.23
Experimental	Viscosity *η*_exp_ (mPa/s)	128.07 ± 0.24	19.23 ± 0.01	45.63 ± 0.20	21.10 ± 0.02
	Density *ρ*_exp_ (g/mL)	0.9152 ± 0.0005	0.9178 ± 0.0001	0.9716 ± 0.0001	0.9675 ± 0.0001
	Surface tension *σ*_exp_ (mN/m)	29.04 ± 0.03	29.41 ± 0.08	31.75 ± 0.01	30.35 ± 0.06
	Refractive index *n*_exp_	1.4670 ± 0.0005	1.4635 ± 0.0008	1.5105 ± 0.0002	1.4970 ± 0.0003
Theoretical	Density *ρ*_L_ (g/mL)	1.1116	1.1063	1.1500	1.1350
	Surface tension *σ*_L_ (mN/m)	25.37	23.25	29.19	32.25
	Refractive index *n*	1.4708	1.4671	1.4795	1.4731

**Table 3:** Error parameter for each parameter.

	$$\mathrm{Percentage}\;\mathrm{Relative}\;\mathrm{Error}\;e_{r}$$			
	Men:Bor	Men:Cam	Thy:Bor	Thy:Cam
Density *ρ*	21.5	20.5	18.4	17.3
Surface tension *σ*	−12.7	−21.0	−8.1	6.3
Refractive index *n*	0.26	0.25	−2.05	−1.59

**Table 4:** Experimental data of the melting temperature (*T*_fus_), enthalpy of fusion (Δ*H*_fus_), of the pure terpenes.

Component	*T*_fus_ (K)	Δ*H*_fus_ (J/mol)
Menthol	309.72	13.62
Thymol	324.31	18.54
Borneol	481.33	7.23
Camphor	452.41	6.32

**Table 5:** Experimental data of the degradation temperature (*T*_d_), the crystallization temperature (*T*_cr_), enthalpy of crystallization (Δ*H*_cr_), and the glass transition temperature (*T*_g_) of the eutectic systems.

Eutectic System	*T*_d_ (K)	*T*_cr_ (K)	Δ*H*_cr_ (J/g)	*T*_g_ (K)
Men:Bor (7:3)	366.02	254.08	21.17	223.03
Men:Cam (3:2)	358.29	239.92	28.60	196.98
Thy:Bor (7:3)	355.04	284.64	0.098	215.17
Thy:Cam (1:1)	370.74	260.69	0.077	194.23

**Table 6:** Predicted eutectic temperatures and molar ratios of the solvent systems.

		Eutectic Systems			
		Men:Bor	Men:Cam	Thy:Bor	Thy:Cam
Ideal	*T*_E, ideal_ (K)	289.8	284.7	309.6	300.1
	*x*_E, ideal_	0.696	0.628	0.722	0.457
UNIFAC	*T*_E, UNIFAC_ (K)	290.0	286.5	300.8	281.7
	*x*_E, UNIFAC_	0.697	0.642	0.645	0.514
COSMO-RS (TZVP)	*T*_E, TZVP_ (K)	289.7	283.4	274.7	166.2
	*x*_E, TZVP_	0.695	0.630	0.515	0.538
COSMO-RS (TZVP-FINE)	*T*_E, TZVPD-FINE_ (K)	290.7	288.1	293.2	237.9
	*x*_E, TZVPD-FINE_	0.704	0.664	0.583	0.547

**Table 7:** Adjusted and optimized parameters for the proposed models of Equations (2) and (3).

Parameter	Value	Units
*σ*_1_	393.4	mN/m
*σ*_2_	−5.3 × 10^−5^	mN/m
*σ*_3_	−3.72 × 10^−2^	mN/m
*σ*_4_	−50.3	mN/m
*σ*_5_	1.132	mN/m
*σ*_6_	108.9	mN/m
*A*_1_	1	mol⋅mL/g^2^
*A*_2_	1	mL⋅m/mol⋅mN
*A*_3_	1	bar⋅mol/g⋅K^0.5^
*A*_4_	1	mol⋅K^0.5^/g
*A*_5_	5.17 × 10^−2^	Dimensionless
*A*_6_	−11.625	mol/g
*A*_7_	2.27 × 10^−3^	bar^−1^
*A*_8_	1.3668	Dimensionless
*A*_9_	25.89	K

## Data Availability

The data presented in this study are available on request from the corresponding author.

## Supplementary Materials

The following are available online. Table S1: The contribution to the critical properties in the modified Lydersen-Joback-Reid method, Table S2: Critical properties of the pure compounds, Table S3: Critical properties of the eutectic mixtures, Table S4: Chemical shifts (δ) of the proton as a function of temperature for each terpene and eutectic system, Table S5: The temperature coefficient Tc of each terpene and eutectic system.

## Sample Availability

Samples of the compounds are not available from the authors.

## Abbreviations

Bor	Borneol
Cam	Camphor
COSMO-RS	Conductor-like Screening Model for Real Solvents
DES	Deep eutectic solvent
DMSO	Deuterated dimethyl sulfoxide
DSC	Differential scanning calorimetry
FTIR	Fourier-transform infrared spectroscopy
Men	Menthol
NMR	Nuclear Magnetic Resonance
SLE	Solid–liquid equilibrium
TGA	Thermogravimetric analysis
Thy	Thymol
UNIFAC	UNIversal Functional Activity Coefficient
Symbols	
*A*_1_–*A*_9_	Optimized parameters for the proposed models
*e*_r_	Percentage relative error
*M*_w_	Molecular weight
*n*	Refractive index
*P*	Pressure
*T*	Temperature
*V*	Volume
*x*	Molar ratio
*γ*	Activity coefficient
Δ*H*	Enthalphy
*η*	Viscosity
*ρ*	Density
*σ*	Surface tension
*σ*_1_–*σ*_6_	Optimized parameters for the proposed models
*ω*	Acentric factor
Subscripts	
c	Critical
coeff	Coefficient
cr	Crystallization
D	Degradation
E	Eutectic
exp	Experimental
g	Glass
i	Compounds i
L	Computed using models
m	Melting/fusion
ref	reference
Superscripts	
R	Residual
L	Computed using models
C	Combinatorial
