# Supplementary material for: Physicochemical Characterization and Simulation of the Solid–Liquid Equilibrium Phase Diagram of Terpene-Based Eutectic Solvent Systems
Source: Molecules. 2021 Mar 23;26(6):1801. doi: 10.3390/molecules26061801 (PMC8004849; doi:10.3390/molecules26061801)
Supplement: Supplementary file 1 [file molecules-26-01801-s001.pdf]

Supplementary material

- **Physical properties computations**

Critical properties of the pure compounds (Modified Lydersen and Joback-Reld model) [1,2]:

$$T_{\mathrm{b},i} = 198.2\ K + \sum n\Delta T_{\mathrm{bM},i} \quad \text{(Eq. S1)}$$

$$T_{\mathrm{c},i} = \frac{T_{b,i}}{0.5703 + 1.0121\ K^{-1} \sum n\Delta T_{\mathrm{bM},i} - 1K^{-2}(\sum n\Delta T_{\mathrm{bM},i})^2} \quad \text{(Eq. S2)}$$

$$P_{\mathrm{c},i} = \frac{M_{\mathrm{w},i}\ \times (1\ bar^3 \frac{mol}{g})}{[0.2573\ bar + \sum nP_{\mathrm{M},i}]^2} \quad \text{(Eq. S3)}$$

$$V_{\mathrm{c},i} = 6.75\ cm^3 + \sum n\Delta V_{\mathrm{M},i} \quad \text{(Eq. S4)}$$

$$\omega_i = \frac{(T_\mathrm{b} - 43\ K)(T_\mathrm{c} - 43\ K)}{(T_\mathrm{c} - T_\mathrm{b})(0.7T_\mathrm{c} - 43\ K)} \log\left(\frac{P_\mathrm{c}}{1.01325\ bar}\right) - \frac{(T_\mathrm{c} - 43\ K)}{(T_\mathrm{c} - T_\mathrm{b})} \log\left(\frac{P_\mathrm{c}}{1.01325\ bar}\right) + \log\left(\frac{P_\mathrm{c}}{1.01325\ bar}\right) - 1 \quad \text{(Eq. S5)}$$

Where T_b is the normal boiling temperature (K), $T_{\mathrm{c},i}$, $P_{\mathrm{c},i}$ and $V_{\mathrm{c},i}$ are the critical temperature (K), pressure (bar) and molar volume (cm^3/mol) of the component i, n is the number of each functional group in the compound, M_w is the molecular weight of compound i (g/mol), and ω_i is the acentric factor. $\Delta T_{bM,\mathrm{i}}$, $\Delta T_{M,\mathrm{i}}$, $\Delta P_{M,\mathrm{i}}$ and $\Delta V_{M,\mathrm{i}}$ are the contribution to the critical properties in the modified Lydersen-Joback-Reid method of compound i and they are computed based on the chemical groups present in each compound, according to the data in Table S1 [1,2].

The critical properties of the eutectic mixtures are obtained based on the Lee-Kesler mixing rules [3]:

$$T_{\mathrm{c},ij} = \sqrt{T_{\mathrm{c},i} \times T_{\mathrm{c},j}} \quad \text{(Eq. S6)}$$

$$V_{\mathrm{c},ij} = \frac{1}{8}(V_{\mathrm{c},i}^{1/3} + V_{\mathrm{c},j}^{1/3})^3 \quad \text{(Eq. S7)}$$

$$V_\mathrm{cm} = y_i^2 V_{\mathrm{c},i} + 2y_i y_j V_{\mathrm{c},ij} + y_j^2 V_{\mathrm{c},j} \quad \text{(Eq. S8)}$$

$$T_\mathrm{cm} = \frac{1}{V_\mathrm{cm}^{0.25}}(y_i^2 V_{\mathrm{c},i}^{0.25} T_{\mathrm{c},ij} + 2y_i y_j V_{\mathrm{c},ij}^{0.25} T_{\mathrm{c},ij} + y_j^2 V_{\mathrm{c},j}^{0.25} T_{\mathrm{c},j}) \quad \text{(Eq. S9)}$$

$$P_\mathrm{cm} = (0.2905 - 0.085\omega_\mathrm{m}) \frac{RT_\mathrm{cm}}{V_\mathrm{cm}} \quad \text{(Eq. S10)}$$

$$\omega_\mathrm{m} = y_i\omega_i + y_j\omega_j \quad \text{(Eq. S11)}$$

Where $T_{\mathrm{c},ij}$ and $V_{\mathrm{c},ij}$ are the critical temperature (K) and volume (cm^3/mol) of the compounds i and j. y_i and y_j are the molar ratio of compounds i and j, respectively, in the eutectic mixture. R is the gas constant (83.14 cm^3·bar/g mole·K). T_cm, P_cm and V_cm are the critical temperature (K), pressure

(bar) and molar volume (cm^3/mol) of the eutectic mixtures and are used to compute the theoretical physical properties (density, surface tension and refractive index) of the eutectic mixtures. Tables S2 and S3 displays the computed values of the each parameter.

Table S 1: The contribution to the critical properties in the modified Lydersen-Joback-Reid method

	**Groups**	**ΔT_{bM} (K)**	**ΔT_M (K)**	**ΔP_M (bar)**	**ΔV_M (cm^3/mol)**
**Without rings**	-CH3	23.58	0.0275	0.3031	66.81
	-CH2-	22.88	0.0159	0.2165	57.11
	>CH-	21.74	0.0002	0.114	45.7
	>C<	18.18	-0.0206	0.0539	21.78
	-CH2	24.96	0.017	0.2493	60.37
	-CH-	18.25	0.0182	0.1866	49.92
	-C<	24.14	-0.0003	0.0832	34.9
	-C-	26.15	-0.0029	0.0934	33.85
	=CH	0	0.0078	0.1429	43.97
	=C-	0	0.0078	0.1429	43.97
	-OH (alcohol)	92.88	0.0723	0.1343	30.4
	-O-	22.42	0.0051	0.13	15.61
	>C=O	94.97	0.0247	0.2341	69.76
	-CHO	72.24	0.0294	0.3128	77.46
	-COOH	169.06	0.0853	0.4537	88.6
	-COO-	81.1	0.0377	0.4139	84.76
	HCOO-	0	0.036	0.4752	97.77
	-O (others)	-10.5	0.0273	0.2042	44.03
	-NH2	73.23	0.0364	0.1692	49.1
	>NH	50.17	0.0119	0.0322	78.96
	>N-	11.74	-0.0028	0.0304	26.7
	-N-	74.6	0.0172	0.1541	45.54
	-CN	125.66	0.0506	0.3697	89.32
	-NO2	152.54	0.0448	0.4529	123.62
	-F	-0.03	0.0228	0.2912	31.47
	-Cl	38.13	0.0188	0.3738	62.08
	-Br	66.86	0.0124	0.5799	76.6
	-I	93.84	0.0148	0.9174	100.79
**With Rings**	-CH2-	27.15	0.0116	0.1982	51.64
	>CH-	21.78	0.0081	0.1773	30.56
	-CH-	26.73	0.0114	0.1693	42.55
	>C<	21.32	-0.018	0.0139	17.62
	-C<	31.01	0.0051	0.0955	31.28

	-O-	31.22	0.0138	0.1371	17.41
	-OH (phenol)	76.34	0.0291	0.0493	-17.44
	>C=O	94.97	0.0343	0.2751	59.32
	>NH	52.82	0.0244	0.0724	27.61
	>N-	0	0.0063	0.0538	25.17
	-N-	57.55	-0.0011	0.0559	42.15

Table S 2: Critical properties of the pure compounds

Compound i	$\Delta T_{bM,i}$ (K)	$\Delta T_{M,i}$ (K)	$\Delta P_{M,i}$ (bar)	$\Delta V_{M,i}$ (cm^3/mol)	$T_{b,i}$ (K)	$T_{c,i}$ (K)	$P_{c,i}$ (bar)	$V_{c,i}$ (cm^3/mol)	ω_i
**Menthol**	315.61	0.1709	2.1991	475.29	513.81	719.56	25.90	482.04	0.509
**Borneol**	292.95	0.1185	1.7583	403.71	491.15	726.35	37.97	410.46	0.404
**Thymol**	327.19	0.1514	1.8910	414.21	525.39	749.90	32.55	420.96	0.511
**Camphor**	311.58	0.1237	1.9841	480.47	509.78	749.46	30.30	487.22	0.342

Table S 3: Critical properties of the eutectic mixtures

Eutectic mixture *i*:*j*	V_{cm} (cm^3/mol)	T_{cm} (K)	P_{cm} (bar)	ω_m
**Men:Bor**	460.16	721.39	32.57	0.48
**Men:Cam**	484.11	731.46	31.77	0.44
**Thy:Bor**	425.98	740.58	35.45	0.53
**Thy:Cam**	453.69	749.49	34.92	0.43

- **SLE phase diagram simulation**

UNIFAC is computed as a function of the combinatorial and residual activity coefficients γ_i^C and γ_i^R. The coefficient γ_i^C is computed based on the Flory-Huggins expression and the Staverman-Guggenheim correction term [4,5] as shown in Eq. S12:

$$\ln \gamma_i^C = 1 - J_i + \ln J_i - 5q_i\left(\ln\frac{\phi_i}{\theta_i} + 1 - \frac{\phi_i}{\theta_i}\right) \quad \text{(Eq. S12)}$$

Where the quantity J_i, the molecule volume and surface area fractions ϕ_i and θ_i, respectively are computed as following:

$$J_i = \frac{\phi_i}{x_i} \quad \text{(Eq. S13)}$$

$$\phi_i = \frac{x_i r_i}{\sum x_i r_i} \quad \text{(Eq. S14)}$$

$$\Theta_i = \frac{x_i q_i}{\sum_j x_j q_j} \quad \text{(Eq. S15)}$$

Where q_i and r_i are the molecular surface area and volume, respectively, and they are obtained as shown in Eq. S16 and S17:

$$q_i = \sum \nu_{\mathrm{k}}^{(i)} R_{\mathrm{k}} \quad \text{(Eq. S16)}$$

$$r_i = \sum \nu_{\mathrm{k}}^{(i)} Q_{\mathrm{k}} \quad \text{(Eq. S17)}$$

Where ν_k corresponds to the number of subgroups of type k in the component i, R_k and Q_k are the subgroup parameters defined as the Van der Waals group volumes and surface areas, respectively. These values are obtained from a database (Dortmund Data Bank) [6].

The coefficient $\gamma_{\mathrm{i}}^{\mathrm{R}}$ is computed based on the Eq. S18:

$$\ln \gamma_i^{\mathrm{R}} = \sum \nu_{\mathrm{k}}^{(i)} \left(\ln \Gamma_{\mathrm{k}} - \ln \Gamma_{\mathrm{k}}^{(i)}\right) \quad \text{(Eq. S18)}$$

Where Γ_{k} and $\Gamma_{\mathrm{k}}^{(i)}$ are the group activity coefficients of the subgroup k in the mixture and in the pure substance i, respectively and they can be computed using Eq. S19 by calculating the sums over all the different groups:

$$\ln \Gamma_{\mathrm{k}} = Q_{\mathrm{k}}\left[1 - \ln\left(\sum_{\mathrm{m}} \Theta_{\mathrm{m}} \psi_{\mathrm{mk}}\right) - \sum_{\mathrm{m}} \frac{\Theta_{\mathrm{m}} \psi_{\mathrm{km}}}{\sum_n \Theta_{\mathrm{n}} \psi_{\mathrm{nm}}}\right] \quad \text{(Eq. S19)}$$

Where ψ_{m} and Θ_{m} correspond to the group interaction parameter and the area fraction of the group m, respectively. They are computed based on Eq. S20 and S22. Θ_{m} is obtained similarly to the Θ_{i} factor calculated in the combinatorial coefficient.

$$\Theta_{\mathrm{m}} = \frac{X_{\mathrm{m}} Q_{\mathrm{m}}}{\sum_n X_{\mathrm{n}} Q_{\mathrm{n}}} \quad \text{(Eq. S20)}$$

Where X_{m} corresponds to the molar fraction of the group m in the mixture and can be obtained as shown in Eq. 17:

$$X_{\mathrm{m}} = \frac{\sum_j \nu_{\mathrm{m}}^{(i)} x_j}{\sum_j \sum_n \nu_{\mathrm{n}}^{(i)} x_j} \qquad \text{(Eq. S21)}$$

The group interaction parameter ψ_{m} depends on the temperature of the system and it is given by:

$$\psi_{\mathrm{m}} = \exp\left(-\frac{a_{\mathrm{mn}}}{T}\right) \qquad \text{(Eq. S22)}$$

The parameter a_{mn} is the binary group interaction parameter and it does not depend on the temperature and it is not symmetric [7]. Therefore, a_{mn} has a different value than a_{nm}. These binary interaction parameters are obtained by fitting a wide range of experimental phase equilibrium data [6].

- **NMR analysis**

Table S 4: Chemical shifts (δ) of the proton as a function of temperature for each terpene and eutectic system

	**Chemical shift *δ* (ppm)**							
***T* (K)**	**Menthol***	**Borneol***	**Thymol***	**Camphor***	**Men:Bor**	**Men:Cam**	**Thy:Bor**	**Thy:Cam**
298	1.4705	1.505	4.8375	1.7442	4.28	3.7	2.5	3.5
303	1.4442	1.4922	4.8032	1.7201	4.18	3.6	2.44	3.41
308	1.4198	1.4702	4.7705	1.6959	4.08	3.51	2.38	3.33
313	1.3966	1.4493	4.7401	1.6711	3.98	3.42	2.32	3.24
318	1.3751	1.4301	4.7111	1.6487	3.86	3.32	2.26	3.16
323	n.d.	n.d.	n.d.	n.d.	3.75	3.22	2.2	3.07

**n.d.: not determined*

Table S 5: The temperature coefficient T_c of each terpene and eutectic system

**Compound**	***T*c (K)**
Menthol	-0.005
Borneol	-0.004
Thymol	-0.006
Camphor	-0.005
Men:Bor	-0.021
Men:Cam	-0.019
Thy:Bor	-0.012
Thy:Cam	-0.017

Reference List:

1. Mirza NR, Nicholas NJ, Wu Y, Kentish S, Stevens GW. Estimation of Normal Boiling Temperatures, Critical Properties, and Acentric Factors of Deep Eutectic Solvents. *J Chem Eng Data*. 2015;60(6):1844-1854. doi:10.1021/acs.jced.5b00046

2. Valderrama JO, Sanga WW, Lazzús JA. Critical properties, normal boiling temperature, and acentric factor of another 200 ionic liquids. *Ind Eng Chem Res*. 2008;47(4):1318-1330. doi:10.1021/ie071055d

3. Labinov SD, Sand JR. An analytical method of predicting Lee-Kesler-Plöcker equation-of-state binary interaction coefficients. *Int J Thermophys*. 1995;16(6):1393-1411. doi:10.1007/BF02083548

4. Prausnitz JM, Lichtenthaler RN, Azevedo EG de. *Molecular Thermodynamics of Fluid-Phase Equilibria.* Prentice Hall PTR; 1999.

5. Voutsas EC, Tassios DP. Analysis of the UNIFAC-Type Group-Contribution Models at the Highly Dilute Region. 1. Limitations of the Combinatorial and Residual Expressions. *Ind Eng Chem Res*. 1997;36(11):4965-4972. doi:10.1021/ie960770c

6. Gmehling J, Li J, Schiller M. A Modified UNIFAC Model. 2. Present Parameter Matrix and Results for Different Thermodynamic Properties. *Ind Eng Chem Res*. 1993;32(1):178-193. doi:10.1021/ie00013a024

7. Tiegs D, Gmehling J, Rasmussen P, Fredenslund A. Vapor—Liquid Equilibria by UNIFAC Group Contribution. 4. Revision and Extension. *Ind Eng Chem Res*. 1987;26(1):159-161. doi:10.1021/ie00061a030
